# Pathways into chronic multidimensional poverty amongst older people: a longitudinal study

**DOI:** 10.1186/s12877-016-0236-z

**Published:** 2016-03-07

**Authors:** Emily J. Callander, Deborah J. Schofield

**Affiliations:** Australian Institute of Tropical Health and Medicine, Discipline of Public Health and Tropical Medicine, Building 41, Douglas Campus, Townsville, QLD 4811 Australia; Faculty of Pharmacy, University of Sydney, Sydney, Australia

**Keywords:** Poverty, Overall health status, Income, Education, Longitudinal analysis

## Abstract

**Background:**

The use of multidimensional poverty measures is becoming more common for measuring the living standards of older people. However, the pathways into poverty are relatively unknown, nor is it known how this affects the length of time people are in poverty for.

**Methods:**

Using Waves 1 to 12 of the nationally representative *Household, Income and Labour Dynamics in Australia* (HILDA) survey, longitudinal analysis was undertaken to identify the order that key forms of disadvantage develop – poor health, low income and insufficient education attainment – amongst Australians aged 65 years and over in multidimensional poverty, and the relationship this has with chronic poverty. Path analysis and linear regression models were used.

**Results:**

For all older people with at least a Year 10 level of education attainment earlier mental health was significantly related to later household income (*p* = 0.001) and wealth (*p* = 0.017). For all older people with at less than a Year 10 level of education attainment earlier household income was significantly related to later mental health (*p* = 0.021).

When limited to those in multidimensional poverty who were in income poverty and also had poor health, older people generally fell into income poverty first and then developed poor health.

The order in which income poverty and poor health were developed had a significant influence on the length of time older people with less than a Year 10 level of education attainment were in multidimensional poverty for. Those who developed poor health first then fell into income poverty spend significantly less time in multidimensional poverty (−4.90, *p* < .0001) than those who fell into income poverty then developed poor health.

**Conclusion:**

Knowing the order that different forms of disadvantage develop, and the influence this has on poverty entrenchment, is of use to policy makers wishing to provide interventions to prevent older people being in long-term multidimensional poverty.

## Background

Numerous studies have been conducted to look at the living standards of older people [1–5]. Consistent with measures of living standards in general, there is a growing recognition that measures of older people’s living standards should be multidimensional [6–10] and not merely focus upon income or economic resources. Contemporary poverty studies routinely focus upon ‘multidimensional poverty’ not just ‘income poverty’, and assess numerous aspects of people’s lives when determining who is in poverty [11–15]. Health and education are common inclusions in such studies. The United Nations Development Program’s original Human Poverty Index included health, education and income, as well as employment, and the Multidimensional Poverty Index, which replaced the Human Poverty Index focuses upon health, education and standard of living [16, 17], the English Indices of Deprivation measure health, disability, education, skills and training, alongside other dimensions such as income and housing [18], and Australia’s Freedom Poverty Measure measures health, education and economic resources [19, 20]. However, there has been very little research measuring the multidimensional poverty status of older people over time using longitudinal data. As a result of this, very little is known about older people’s movement into multidimensional poverty and in what order their multiple forms of disadvantage develop.

Knowing what pathways older people took into multidimensional poverty would give a more holistic view of living standards – providing insights into how some older people come to be multidimensionally poor and potentially identifying points for effective intervention to prevent multidimensional poverty. This paper aims to assess the different pathways into multidimensional poverty amongst older people using the Freedom Poverty Measure, a multidimensional measure of poverty developed specifically for the Australian population. It assesses health, education and income to determine an individual’s poverty status [19, 20]. In constructing the Freedom Poverty Measure, income, health and education were chosen as the dimensions of disadvantage for their demonstrated impact on the living standards of individuals [21–30]. This measure is discussed in further detail below. The paper aims to identify the order in which poor health and income poverty are generally developed amongst those who are multidimensionally poor and whether this relationship is different for those with a higher level of education attainment. The paper focuses upon those aged 55 years and over in Australia.

## Methods

### Data set sampling and weighting

This is a longitudinal study utilising the Household Income and Labour Dynamics in Australia (HILDA) Survey focusing on the Australian population aged 55 years and over in 2001. The HILDA survey is a longitudinal survey of private Australian households conducted annually since 2001. The data are nationally representative of the Australian population living in private dwellings and aged 15 years and over [31]. The survey sampling unit for Wave 1 was the household, with all members of the household being part of the sample that would be followed over the life of the survey – however, only those aged 15 and over had detailed individual information recorded. Household sampling was conducted in a three-stage approach. Initially 488 Census Collection Districts (each containing 200 to 250 households) were selected, then within each district 22 to 34 dwellings were selected, finally up to three households within each dwelling were selected to be part of the sample. This paper focused upon the continuing person sample from Waves 1 to 12.

The HILDA survey is available, upon application from the Australian Department of Social Services (https://www.melbourneinstitute.com/hilda/data/).

### Income, health, education and poverty measures

The Freedom Poverty Measure is based upon the capabilities theory of Nobel prize winning economist Amartya Sen [32]. People in poverty are seen to have poor living standards because they do not have the capabilities that allow them to participate, engage and function in society [19]. Rather than attempting to measure living standards, which is an intangible concept and varies between individual’s due to differences in tastes and preferences, the Freedom Poverty Measure focuses on measuring universal capabilities that allow people to build the type of living standards they have cause to desire. The capability indicators included in the Freedom Poverty Measure are income, health and education attainment. An individual is considered to be in multidimensional poverty if they are in income poverty and have either poor health or an insufficient level of education attainment, thus measuring the joint distribution of these factors. These three factors were selected as they were seen to be key factors that influence an individual’s ability to participate fully within all aspects modern Australian society [19].

Income was based upon total regular household income, which was composed of regular private income (wages and salary, business income, investment income, and private pensions and transfers), Australian government public transfers (government income support payments and other government payments, such as family or carer payments), other public payments such as scholarships, and foreign pensions. This total income was then equivalised for the number and age of household members using the OECD-modified equivalence scale [33]. The cut-off point for having low income, or not, was having an equivalised income less than 50 % of the median equivalised income for the Australian population of all ages.

Health status was measured using the Physical Component Summary (PCS) and Mental Component Summary (MCS) scores from the SF-36 health scale [34], which was available from the HILDA dataset. The PCS was used to measure physical health and MCS was used to measure mental health. Those with poor health had a PCS or MCS less than 75 % of the average for their age group.

Education attainment was measured based upon a person’s highest level of education attainment. Those who had achieved lower than Year 10 (Year 9, Year 8, Year 7, primary education only, Certificate I, Certificate II, or certificate undefined) were considered to have an insufficient level of education attainment [35].

This paper also assesses the length of time an individual is multidimensionally poor for. In this paper, those in chronic multidimensional poverty were classified as those who were in freedom poverty for four years or longer. This time frame is based upon the reduced probability of exiting multidimensional poverty after four years (author’s analysis, under review).

### Ethics approval

As no research was undertaken on human subjects, ethics approval was not required. The data used in this analysis was approved by the Commonwealth Department of Social Services.

### Statistical analysis

To estimate the longitudinal relationship between education, health and income, we fitted cross-lagged path analysis models, which assessed the relationship in both directions (income affecting health and also health affecting income). Income was measured using total equivalised household income as a continuous variable, and physical and mental health were measured using the SF-36 PCS and MCS scores as continuous variables. Wealth was also included due to its close relationship with income and was measured using total equivalised household wealth as a continuous variable. Three time points were included – 2002, 2006 and 2010 as wealth was only included in these waves. The goodness-of-fit of the models was assessed by the chi-square test, CFI, TLI and root mean square error of approximation (RMSEA). Models were tested separately for those with a sufficient level of education attainment (Year 10 and above) and for those with an insufficient level of education attainment. Both models were controlled for age and sex. As the data was collected using the same survey instruments in successive years, the error terms for physical health, mental health, income and wealth were allowed to co-vary.

To analyse the order in which forms of disadvantage were developed for those in income poverty and with poor health, those in multidimensional poverty in 2009 were identified along with those who were in chronic poverty or went on to be in chronic poverty by 2012. Of those in multidimensional poverty in 2009, the order in which the forms of disadvantage were developed was then identified.

Separate linear regression models were constructed to assess the relationship between pathway into multidimensional poverty and the length of time in multidimensional poverty, adjusting for age, sex, area of residence (major city, inner regional area, outer regional area), employment status (employed full time, employed part time, unemployed, not in the labour force), marital status, home ownership, and equivalised household wealth. Those with unknown pathways into multidimensional poverty (they had fallen into freedom poverty before 2001) were excluded from the analysis. Models were fitted separately for those with a sufficient level of education attainment and those with an insufficient level of education attainment.

The analyses were performed using IMB SPSS AMOS 22.0.0 and SAS V9.4. Statistical significance was set at the 5 % level.

## Results

This study focuses on the 1742 records of people who responded to each wave of the HILDA survey between Waves1 and 12 and were aged 55 and over in Wave 1, representing 2,842,400 people in the population in 2001. The mean age in 2001 was 64.5 (SD = 7.1) and 55 % were female.

**Fig. 1:**
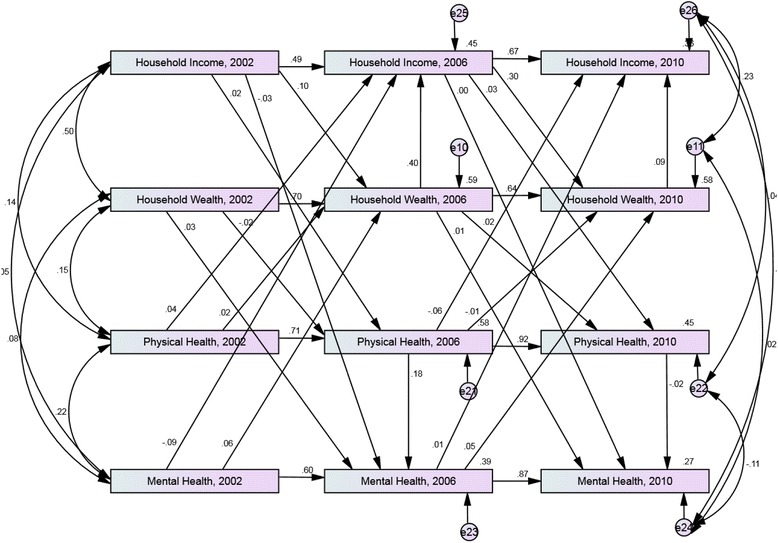
Cross-lagged model of equivalised household income, household wealth, physical health and mental health between 2002 and 2010. Sample with *sufficient level of education attainment*, aged 55 and over in 2001. Model adjusted for age and sex. Standardized estimates shown

The cross-lagged path analysis model of equivalised annual household income, equivalised household wealth, physical health and mental health between 2002 and 2010 for those with a sufficient level of education attainment is shown in Fig. 1. The model showed a good fit *X*^*2*^ (10) = 7.5, *p* = 0.680; TLI = 1.00; CFI = 1.00; RMSEA = 0.00. Table 1 shows the magnitude of the association between mental health in 2002 and equivalised household income in 2006 (*p* = 0.001), mental health in 2002 and equivalised household wealth in 2006 (*p* = 0.017), and mental health in 2006 and equivalised household income in 2010 (*p* = 0.066). There was no evidence of a significant relationship between earlier household income or wealth and later physical health or mental health, and earlier physical health and later household income or wealth.

**Table 1:** Standardised direct and indirect effects between equivalised household income, physical health status and mental health status from 2002 to 2010. Sample with sufficient level of education attainment, aged 55 and over in 2001

Path	Standardised direct effect	Standardised indirect effect	Total effect	*p*-value
Mental Health, 2002 > Equivalised household Income, 2006	−0.092	0.024	−0.061	0.001
Mental Health, 2002 > Equivalised household wealth, 2006	0.061	-	0.061	0.017
Mental Health, 2006 > Equivalised household Income, 2010	0.009	0.005	0.014	0.066

**Fig. 2:**
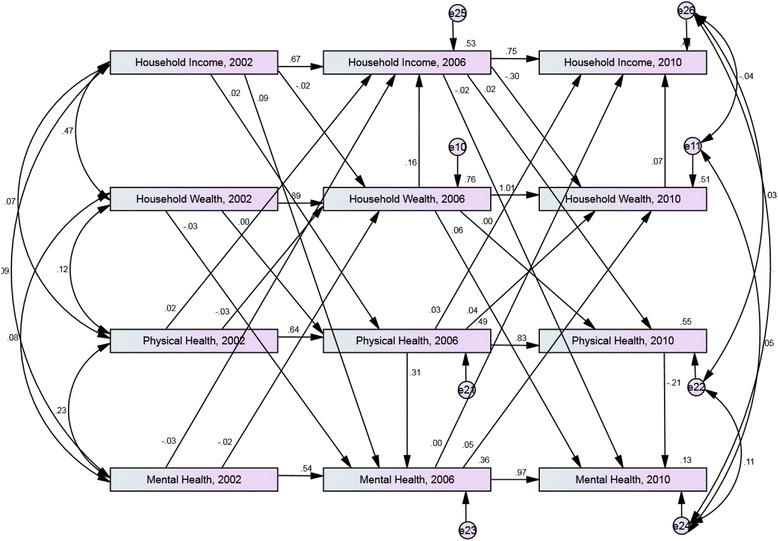
Cross-lagged model of equivalised household income, household wealth, physical health and mental health between 2002 and 2010. Sample with *insufficient level of education attainment*, aged 55 and over in 2001. Model adjusted for age and sex. Standardized estimates shown

The cross-lagged path analysis model of equivalised annual household income, equivalised household wealth, physical health and mental health between 2002 and 2010 for those with an insufficient level of education attainment is shown in Fig. 2. The model showed a good fit *X*^*2*^ (10) = 9.7, *p* = 0.465; TLI = 1.00; CFI = 1.00; RMSEA = 0.00. Table 2 shows the magnitude of the association between equivalised household income in 2002 and mental health in 2006, which was shown to be significant (*p* = 0.021). There was no evidence of a significant relationship between earlier household income or wealth and later physical health, and earlier physical or mental health and later income or wealth.

**Table 2:** Standardised direct and indirect effects between equivalised household income, physical health status and mental health status from 2002 to 2010. Sample with insufficient level of education attainment, aged 55 and over in 2001

Path	Standardised direct effect	Standardised indirect effect	Total effect	*p*-value
Equivalised household Income, 2002 > Mental Health, 2006	0.087	0.006	0.093	0.021

### Pathways into multidimensional poverty

In 2009, 27 % of older Australians were in multidimensional poverty. Of the people in multidimensional poverty in 2009, 22 % had an insufficient level of education attainment and were in income poverty, 53 % had poor health and were in income poverty, and 25 % had all three forms of disadvantage: poor health, an insufficient level of education attainment and were in income poverty.

Of those with poor health who were in income poverty but had a sufficient level of education attainment, 30 % developed poor health first and then fell into low income afterwards, 45 % were in low income first and then developed poor health, 21 % fell into low income and developed poor health in the same year, and 4 % developed poor health and low income before Wave 1 of HILDA so it is unknown which developed first (Table 3).

**Table 3:** Pathways into multidimensional poverty between 2001 and 2009. Sample with sufficient level of education attainment, aged 55 and over in 2001, who were in multidimensional poverty in 2009

1^st^ form of disadvantage	2^nd^ form of disadvantage	Number in population in 2009	Proportion in that type of disadvantage combination	Mean length of time in poverty (years)	Proportion in chronic poverty
Poor health	Low income	50 000	30 %	2.2	22 %
Low income	Poor health	75 700	45 %	2.5	16 %
Low income and poor health simultaneously		36 300	21 %	2.9	33 %
Unknown		7 200	4 %	10	100 %

Table 4 shows the order in which poor health and income poverty were developed by those with all three forms of disadvantage: poor health, income poverty and a low level of education attainment. Of these people, 24 % developed poor health first and then fell into income poverty afterwards, 51 % fell into income poverty first and then developed poor health, 16 % fell into income poverty and developed poor health in the same year, and 7 % developed poor health and income poverty before Wave 1 of HILDA so it is unknown which developed first (Table 4).

**Table 4:** Pathways into multidimensional poverty between 2001 and 2009. Sample with insufficient level of education attainment, aged 55 and over in 2001, who were in multidimensional poverty in 2009

1^st^ form of disadvantage	2^nd^ form of disadvantage	Number	% of those with three forms of disadvantage	Mean length of time in poverty (years)	Proportion in chronic poverty
Poor health	Low income	45 000	24 %	4.4	49 %
Low income	Poor Health	97 300	51 %	9.3	98 %
Poor health and low income simultaneously		30 400	16 %	2.9	30 %
Unknown order		13 500	7 %	10	100 %

### Pathways into poverty and chronic poverty

Of those with poor health who were in income poverty but had a good level of education attainment, 16 % of those who fell into income poverty first and then developed poor health were in chronic poverty (in multidimensional poverty for four consecutive years), and the average length of time spent in multidimensional poverty was 2.5 years (Table 3). Twenty-two percent of those who developed poor health first and then fell into income poverty second were in chronic poverty and the average length of time spent in multidimensional poverty was 2.2 years; 33 % of those who fell into income poverty and developed poor health in the same year were chronically poor and the average length of time spent in multidimensional poverty was 2.9 years (Table 3).

Table 5 shows the regression model of length of time spent in multidimensional poverty for those with a sufficient level of education attainment. After adjusting for sex, age, place of residence, employment status, marital status, home ownership and amount of household wealth, there was no significant difference in the amount of time spent in multidimensional poverty between those who fell into income poverty first and those who developed poor health first (*p* = 0.8403). Similarly, there was no significant difference in the amount of time spent in multidimensional poverty between those who fell into income poverty and developed poor health in the same year and those who developed fell into income poverty first (*p* = 0.7214).

**Table 5:** Model of length of time in multidimensional poverty between 2001 and 2012 based upon the order in which poor health and income poverty were developed. Sample with sufficient level of education attainment, aged 55 and over in 2001, who were in multidimensional poverty in 2009

Estimated regression coefficients				
Parameter	Estimate	Standard error	*t* value	*p* value
Intercept	4.89^a^	2.34	2.08	0.0398
Poor health then income poverty	−0.08	0.40	−0.20	0.8403
Poor health and income poverty in same year	0.18	0.50	0.36	0.7214
Male	0.53	0.41	1.29	0.1987
Age	−0.04	0.03	−1.08	0.2848
Lives in inner regional area	0.51	0.63	0.81	0.4177
Lives in outer regional area	−0.60	0.64	−0.94	0.3498
Employed part time	−0.37	1.45	−0.26	0.7979
Unemployed	2.02	1.24	1.63	0.1066
Not in the labour force	0.23	1.16	0.20	0.8418
Married	−0.83^a^	0.39	−2.12	0.0369
Owns own home	0.87	0.47	1.87	0.0643
Household wealth	−0.00^a^	0.00	−2.98	0.0036

^a^Significant at the 0.05 level; ^b^Significant at the 0.001 level

Dependant variable = length of time in multidimensional poverty

R^2^ = 0.2018

Amongst those with all three forms of disadvantage the proportion in chronic poverty and length of time in multidimensional poverty was generally much higher. Of those who fell into income poverty first and then developed poor health, 98 % were chronically poor and the average length of time spent in multidimensional poverty was 9.3 years; whereas 49 % of those who developed poor health first then fell into income poverty second were chronically poor and the average length of time spent in multidimensional poverty was 4.4 years (Table 4).

Table 6 shows the regression model of length of time spent in multidimensional poverty for those with an insufficient level of education attainment. After adjusting for sex, age, place of residence, employment status, marital status and home ownership, those who developed poor health first spend significantly less time in multidimensional poverty than those who fell into income poverty first (*p* < .0001). Similarly, those who fell into income poverty and developed poor health in the same year also spent significantly less time in multidimensional poverty than those who developed fell into income poverty first (*p* < .0001).

**Table 6:** Model of length of time in multidimensional poverty between 2001 and 2012 based upon the order in which poor health and income poverty were developed. Sample with insufficient level of education attainment, aged 55 and over in 2001, who were in multidimensional poverty in 2009

Estimated regression coefficients				
Parameter	Estimate	Standard error	*t* value	*p* value
Intercept	23.60^a^	7.68	3.07	0.0027
Poor health then income poverty	−4.90^a^	0.72	−6.80	<.0001
Poor health and income poverty in same year	−6.61^a^	0.72	−9.14	<.0001
Male	0.12	0.71	0.17	0.8658
Age	0.09	0.05	1.72	0.0883
Lives in inner regional area	0.19	0.67	0.28	0.7808
Lives in outer regional area	0.14	1.00	0.14	0.8864
Employed part time	−24.63^a^	8.07	−3.05	0.0029
Unemployed	0.00	0.00	.	.
Not in the labour force	−19.00^b^	7.56	−2.51	0.0136
Married	−1.03	0.68	−1.52	0.1322
Owns own home	0.39	0.69	0.57	0.5716
Household wealth	−0.00^a^	0.00	−2.96	0.0039

^a^Significant at the 0.001 level; ^b^Significant at the 0.05 level

Dependant variable = length of time in multidimensional poverty

R^2^ = 0.5790

## Discussion

The results of this study have shown that the relationship between health and income over time is complex, with earlier mental health affecting later income and wealth amongst those with a better education; whereas earlier income effected later mental health for those with lower levels of education attainment. The results also revealed that there are numerous pathways into multidimensional poverty. For people with poor health who were in income poverty, slightly more people fell into income poverty first and then developed poor health. The finding that low income precedes poor health is in line with the social determinants of health theory, which advocates that health is influenced by income [36]. However, most of the studies that support this theory are based upon cross-sectional analysis and so have been unable to establish the order in which poor health and low income were developed [37–39]. Only a small number of studies have utilised longitudinal data to look at poor health and income poverty. It has been noted in a recent systematic review, that numerous longitudinal studies looking at the influence of income on health found no significant relationship, or where a significant relationship was found, the effect of income on health was very small [40]. The results of this study have also shown that a sizable proportion of people experience the inverse relationship – their health initially declines and then they fall into income poverty. This is the first study to document this relationship for older people.

The results have also shown that the order in which forms of disadvantage are developed amongst older people does influence the length of time people are in multidimensional poverty for, however we only found evidence for this amongst people with lower levels of education attainment. Those who fell into income poverty and then developed poor health were likely to be in poverty for longer than those who developed poor health first. It is recognised that older people are not financially prepared for illness, with many having to utilise or sell accumulated assets and capital [41, 42]. Older people who were already in income poverty may not have had the financial assets to access the same amount of healthcare that people who were financially better off may have been able to, resulting in more severe and persistent health outcomes.

This study does have a number of limitations that need to be considered. Health status was measured based upon SF-36 which is a self-assessed measure of health status, and no clinical diagnosis of chronic health conditions was able to be included in the study. Furthermore, the HILDA survey is a household survey and as such older people in nursing homes, aged care facilities, hospitals and similar institutions were not included in the sample. These people are likely to have poorer health than people still living in their own homes, which may have biased the results.

## Conclusions

Despite these limitations, knowing the order that different forms of disadvantage develop amongst older people, and the influence this has on poverty entrenchment, is of use to policy makers wishing to provide interventions to prevent the number of older people within the population being in long-term multidimensional poverty.

## Abbreviations

HILDA: Household Income and Labour Dynamics in Australia survey
PCS: Physical Component Summary
MCS: Mental Component Summary
